# Long-Term Effects of Spironolactone on Kidney Function and Hyperkalemia-Associated Hospitalization in Patients with Chronic Kidney Disease

**DOI:** 10.3390/jcm7110459

**Published:** 2018-11-21

**Authors:** Chen-Ta Yang, Chew-Teng Kor, Yao-Peng Hsieh

**Affiliations:** 1 Department of Internal Medicine, Changhua Christian Hospital, Changhua 50006, Taiwan; 149054@cch.org.tw (C.-T.Y.); 179297@cch.org.tw (C.-T.K.); 2 School of Medicine, Kaohsiung Medical University, Kaohsiung 80708, Taiwan; 3 School of Medicine, Chung Shan Medical University, Taichung 40201, Taiwan

**Keywords:** chronic kidney disease (CKD), end-stage renal disease (ESRD), major adverse cardiovascular events (MACE), mortality, spironolactone

## Abstract

Background: Spironolactone, a non-selective mineralocorticoid receptor antagonist, can protect against cardiac fibrosis and left ventricular dysfunction, and improve endothelial dysfunction and proteinuria. However, the safety and effects of spironolactone on patient-centered cardiovascular and renal endpoints remain unclear. Methods: We identified predialysis stage 3–4 chronic kidney disease (CKD) patients between 2000 and 2013 from the Longitudinal Health Insurance Database 2005 (LHID 2005). The outcomes of interest were end-stage renal disease (ESRD), major adverse cardiovascular events (MACE), hospitalization for heart failure (HHF), hyperkalemia-associated hospitalization (HKAH), all-cause mortality and cardiovascular mortality. The Fine and Gray sub-distribution hazards approach was adopted to adjust for the competing risk of death. Results: After the propensity score matching, 693 patients with stage 3–4 CKD were spironolactone users and 1386 were nonusers. During the follow-up period, spironolactone users had a lower incidence rate for ESRD than spironolactone non-users (39.2 vs. 53.69 per 1000 person-years) and a higher incidence rate for HKAH (54.79 vs. 18.57 per 1000 person-years). The adjusted hazard ratios for ESRD of spironolactone users versus non-users were 0.66 (95% CI, 0.51–0.84; *p* value < 0.001) and 3.17 (95% CI, 2.41–4.17; *p* value < 0.001) for HKAH. A dose-response relationship was found between spironolactone use and risk of ESRD and HKAH. There were no statistical differences in MACE, HHF, all-cause mortality and cardiovascular mortality between spironolactone users and non-users. Conclusion: Spironolactone represented a promising treatment option to retard CKD progression to ESRD amongst stage 3–4 CKD patients, but strategic treatments to prevent hyperkalemia should be enforced.

## 1. Introduction

Chronic kidney disease (CKD), having an increasing prevalence rate of about 10% worldwide as a result of longevity and ongoing epidemic of diabetes mellitus (DM), is an emerging global health concern and associated with the risk of premature cardiovascular disease (CVD) and mortality [1,2]. The above-expected cardiovascular risk may be attributed to some unique features in CKD, comprising anemia, endothelial dysfunction, oxidative stress, abnormalities in calcium and phosphorus metabolism and metabolic acidosis. These derangements can further accelerate coronary calcification, coronary atherosclerosis, left ventricular hypertrophy and eventually heart failure [3].

Among the available treatment options to retard renal function decline, blockade of renin-angiotensin-aldosterone system (RAAS) by angiotensin-converting enzyme inhibitor (ACEI) or angiotensin receptor blocker (ARB) has been widely used for their potentially beneficial effects on renal and cardiovascular outcomes in both diabetic and non-diabetic patients [4,5]. The renoprotective impact takes effects mainly as a result of reduction of intra-glomerular pressure and inhibition of angiotensin-II induced mesangial cell proliferation and fibrosis [6]. The phenomenon of aldosterone synthesis escape had been proposed to explain that neither ACEI nor ARB can completely abrogate or retard the progression of kidney disease [7]. Therefore, mineralocorticoid receptor antagonists (MRA) have been evaluated for their promising role in conjunction with ACEI or ARB afterwards.

Several studies on the effects of MRA were conducted amongst CKD patients in the past one decade. A 2014 meta-analysis, updated from a 2009 meta-analysis, included 1549 patients of 27 studies and demonstrated that addition of MRA to ACEI or ARB (or both) in mild to moderate CKD patients resulted in the reduction of proteinuria and blood pressure, but precluded conclusive evidence regarding the risk of major cardiovascular events or end-stage renal disease (ESRD) [8]. The higher risk of hyperkalemia and gynecomastia limits its prescription in clinical practice. A later meta-analysis by Ng et al. in 2015 also drew similar conclusions, but the impact of MRA on CVD morbidity and mortality was unable to evaluate due to insufficiency data [9]. The major flaws in the studies of these meta-analyses were relatively small patient numbers and short follow-up period relatively. Spironolactone, a non-selective MRA, can protect against cardiac fibrosis and left ventricular dysfunction, and improve endothelial dysfunction and proteinuria [10]. Therefore, we conducted this retrospective cohort study using representative national data to evaluate the effects of spironolactone on all-cause mortality, CVD mortality, ESRD, hospitalization for heart failure (HHF), major adverse cardiovascular events (MACE) and hyperkalemia-associated hospitalization (HKAH) in predialysis stage 3–4 CKD patients.

## 2. Experimental Section

### 2.1. Data Source

The National Health Insurance Research Database (NHIRD) of Taiwan, which included the healthcare utilization data covering 99% of Taiwanese population, was released for the purpose of scientific research. The Longitudinal Health Insurance Database 2005 (LHID 2005), which contained the information of 1 million people, was randomly selected from the NHIRD and was used as the research database for our study. LHID 2005 contained the comprehensive de-identified healthcare information on demographic, outpatient visit, inpatient care, prescription drugs and medical procedures from 1996 to 2013. The Bureau of Taiwan Health Insurance adopted the International Classification of Diseases, Ninth Revision, Clinical Modification (ICD-9-CM) codes for medical coding. The drugs were classified according to the Anatomical Therapeutic Chemical (ATC) classification system. This study was approved by the Institutional Review Board (IRB) of the Changhua Christian Hospital (approval number CCH-IRB 180717) and conducted in accordance with the declaration of Helsinki. The written informed consents were waived for a retrospective study in Taiwan.

### 2.2. Study Design and Participants

From 1996 to 2013, we identified 21,693 patients with CKD who were defined as having at least one record of a CKD diagnostic code made by a nephrologist per month for at least three consecutive months. We used a 4-year look-back (1996–1999) period to determine patients with incident CKD by excluding pre-existing CKD diagnosis. In addition, incident CKD patients who had incomplete demographic data; were aged <18 years, >100 years; had advanced CKD (stage 5), acute kidney injury, end-stage renal disease; or had a follow-up time of <90 days were also excluded. The remaining incident CKD patients represented moderate to severe (stage 3–4) CKD patients. Among them, patients who had received spironolactone within 90 days after CKD diagnosis were considered as spironolactone users, whereas the remaining patients were considered as non-users. Because we used drug prescription information within 90 days after CKD diagnosis to ascertain spironolactone use, the 91st day after CKD diagnosis was defined as the index date.

### 2.3. Outcome Measures and Relevant Variables

The outcomes of interest were ESRD requiring renal replacement therapy, MACE, HHF, HKAH, all-cause mortality and CVD mortality. MACE was the composite of acute myocardial infarction (ICD-9 code 410) and ischemic stroke (ICD-9 code 433–436). We defined HHF and HKAH as the first listed ICD-9 code 428 or 276.7 of the discharge diagnoses during the follow-up period, respectively. Causes of death were defined by either the main diagnosis for in-hospital death or the first discharge diagnosis of the last hospitalization within three months before death outside the hospital. The end of study was on 31 December 2013. Death was considered a competing event for the occurrence of ESRD, MACE, HHF, and HKAH.

Potential relevant confounders included demographic (age, gender, monthly income and geographic location), comorbid diseases defined by at least three corresponding diagnostic codes within 1 year before index date, and long-term medications use, such as anti-hypertensive drugs, anti-diabetic medication, statin, aspirin, NSAIDs and NaHCO3. Charlson comorbidity index scores (CCIs) were used to measure the severity of baseline comorbidities.

### 2.4. Statistical Analyses

Data were presented as mean ± standard deviation (SD) for continuous variables and number (percentage) for categorical variables. The distribution of patients’ characteristics between spironolactone users and non-users was compared using chi-squared tests and t-test for categorical and continuous variables, respectively. The propensity score was calculated by performing non-parsimonious multivariate logistic regression using all the patient’s characteristic variable (Table 1). The resulting propensity score indicated the probability of being treated with spironolactone and was used for matching processes. The spironolactone non-users were matched by propensity score to the spironolactone users in 2:1 ratio. We used the nearest-neighbor algorithm with a caliper of 0.1 standard deviation (SD) units to construct matched pairs, with the assumption that the proportion of 1.0 is perfect.

We calculated the incidence rate (per 1000 person-years) for outcomes of interest. The cumulative incidence of study outcomes over time between spironolactone users and non-users was estimated using modified Kaplan-Meier method (Fine and Gray sub-distribution hazards approach) and compared using Gray’s tests. The Cox proportional hazards regression models were performed to assess the association between spironolactone use and outcomes of interest, with hazard ratio (HR) and 95% confidence interval (CI) to determine the statistical significance. Because death would impede the occurrence of other study outcomes, the Fine and Gray sub-distribution hazards approach was adopted to adjust for the competing risk of death.

To determine the dose-response association, we estimated the risk of clinical outcomes for spironolactone users versus non-users according to the prescribed daily dose (<12.5, 12.5–25, or ≥25 mg) and the cumulative defined daily dose (DDD) during the 90-day exposure period (≤30 or >30 DDD). The spironolactone DDD defined by WHO is 75 mg. Subgroup analyses were performed to assess the effect modification and we tested the association between spironolactone use and study outcomes of statistical significance in different patient groups. Furthermore, we performed a series of sensitivity analyses to obtain a robust result. First, we evaluated misclassification bias by re-defining spironolactone use at interval of 60, 120 and 180 days after CKD diagnosis. Second, we excluded patients in control cohort who received spironolactone during the follow-up period. Third, an as-treat (AT) model was performed to examine the results when patients who switched to receive or discontinue spironolactone were censored. Fourthly, we also checked the cohort effects by dividing all the study patients into two exposure year periods. Lastly, we re-ran the multivariate regression analysis using the raw data before propensity score matching (spironolactone users = 785, non-users = 13,884). All statistical analyses were performed using R language and SPSS statistical software, version 20.0 (SAS 9.4 software (SAS Institute Inc., Cary, NC, USA)). Two-tailed *p*-value < 0.05 was considered as significant.

## 3. Results

### 3.1. Characteristics of Patients

**Figure 1:**
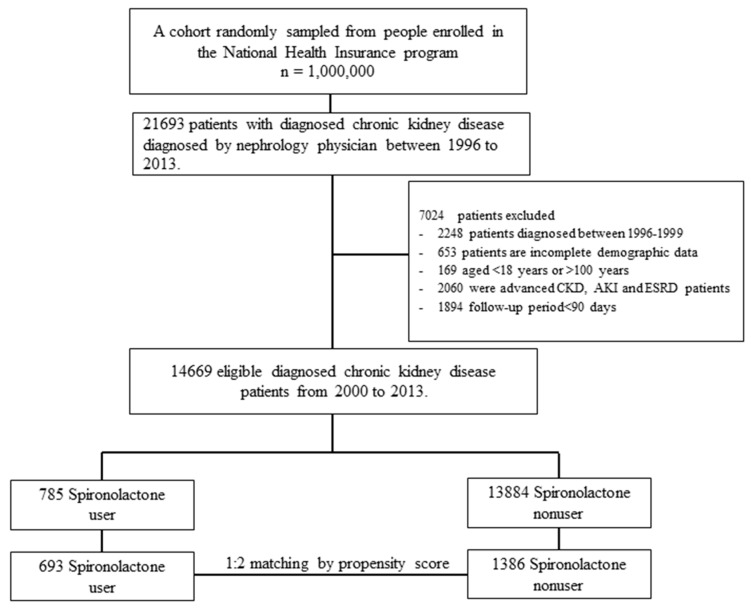
Flowchart of patient selection processes for stage 3–4 chronic kidney disease CKD with or without spironolactone use.

A flowchart of the subjects’ selection process was shown in Figure 1. After excluding CKD patients with advanced (stage 5) CKD or dialysis-dependent ESRD, a total of 14,699 patients with moderate to severe CKD (stage 3–4) were enrolled from LHID 2005 between 2000 and 2013. After the propensity score matching in a 1:2 ratio, 693 patients were spironolactone users and 1386 were nonusers. The mean follow-up time for spironolactone users and non-users were 3.57 ± 3.2 and 3.24 ± 3.23 years, respectively. Table 1 shows the baseline characteristics of study patients stratified by spironolactone use before and after propensity score matching. After matching, there was no significant difference in the distribution of all baseline characteristics between spironolactone users and non-users. The spironolactone users had a reduced prevalence of ESRD (12.7% vs. 19.19%, *p* value < 0.001) but an increased prevalence of HKAH (17.75% vs. 6.64%, *p* value < 0.001) compared with the spironolactone non-users. However, there was no significant difference in prevalence of MACE, HHF and mortality.

### 3.2. Long-Term Risk of Incident ESRD

**Figure 2:**
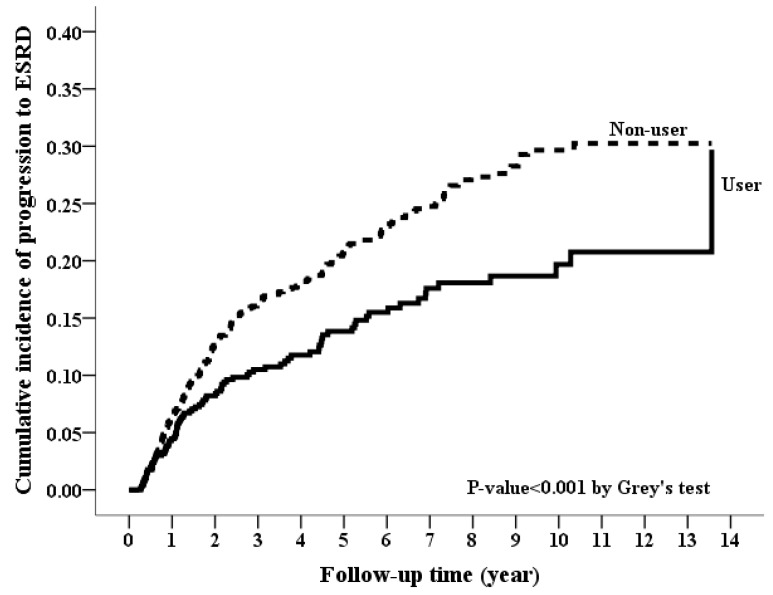
Cumulative incidence rate of progression to end-stage renal disease between spironolactone users and non-users. (*p*-value < 0.001, Grey’s test).

During the follow-up period, spironolactone users had a lower incidence rate for ESRD than spironolactone non-users (39.2 vs. 53.69 per 1000 person-years) (Table 2). Kaplan-Meier curves showed a significantly lower cumulative incidence of ESRD for spironolactone users (*p* value < 0.001 in Figure 2). In Cox’s competing risk model analyses, the spironolactone users had a lower risk of ESRD (crude HR, 0.65; 95% CI, 0.51–0.83; *p* value < 0.001) compared with the spironolactone non-users. After adjustment for all confounders, the association was unchanged (adjusted HR [aHR], 0.66; 95% CI, 0.51–0.84; *p* value < 0.001). An inverse dose-response relationship was found between spironolactone use and risk of ESRD. Compared with CKD patients not taking spironolactone, patients who took prescribed daily dose of spironolactone ≥25 mg, 12.5–25 mg and <12.5 mg had significantly a lower risk of ESRD with aHR of 0.57 (0.35–0.91), 0.59 (0.39–0.91), and 0.73 (0.52–1.00), respectively (*p* value = 0.0057 for trend in Table 3). Similarly, those who took cumulative doses of spironolactone >30 DDD and ≤30 DDD had a significantly lower risk of ESRD with aHR (95% CI) of 0.60 (0.37–0.97) and 0.68 (0.52–0.89), respectively (*p* value = 0.0038 for trend).

### 3.3. Long-Term Risk of Hyperkalemia-Associated Hospitalization

**Figure 3:**
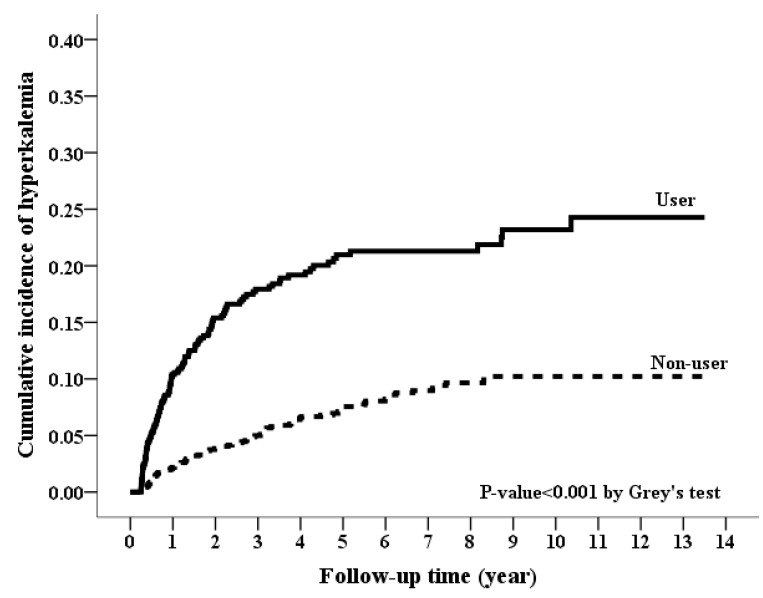
Cumulative incidence rate of hyperkalemia-associated hospitalization between spironolactone users and non-users. (*p*-value < 0.001, Grey’s test).

During the follow-up period, spironolactone users had a higher incidence rate for HKAH than spironolactone non-users (54.79 vs. 18.57 per 1000 person-years) (Table 2). Spironolactone users also had significantly a higher cumulative incidence for HKAH (*p* value < 0.001 in Figure 3). In both thecrude and adjusted models, the spironolactone users still had a significantly higher risk of HKAH (crude HR, 2.98; 95% CI, 2.28–3.90; *p* value < 0.001; aHR, 3.17; 95% CI, 2.41–4.17; *p* value < 0.001) than spironolactone non-users (Table 2). In the dose-response effect, the higher dose of spironolactone use was consistently associated with a higher risk for HKAH in both of the defined doses (both *p* values < 0.001 for trend in Table 3).

### 3.4. Long-Term Risk of MACE, Hospitalization for Heart Failure and Mortality

**Figure 4:**
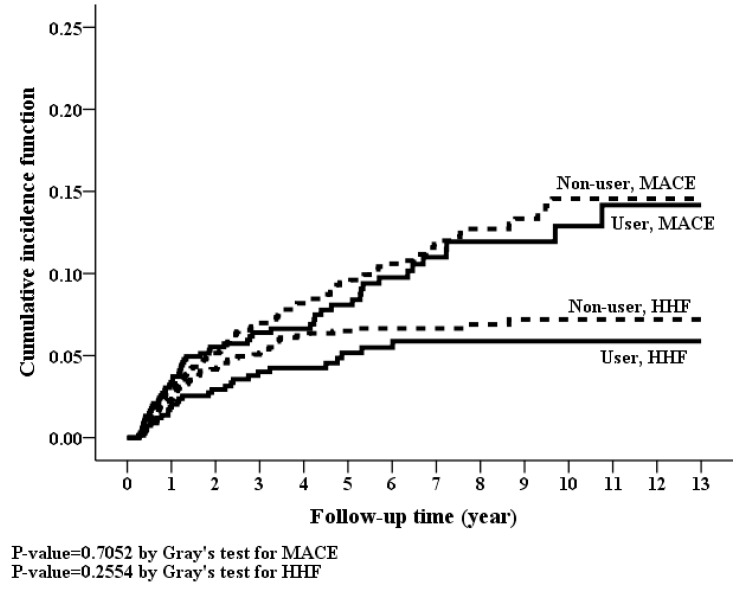
Cumulative incidence rate of hospitalization for heart failure (*p*-value = 0.2554, Grey’s test) and major adverse cardiovascular events (*p*-value = 0.7052, Grey’s test) between spironolactone users and non-users between spironolactone users and non-users.

**Figure 5:**
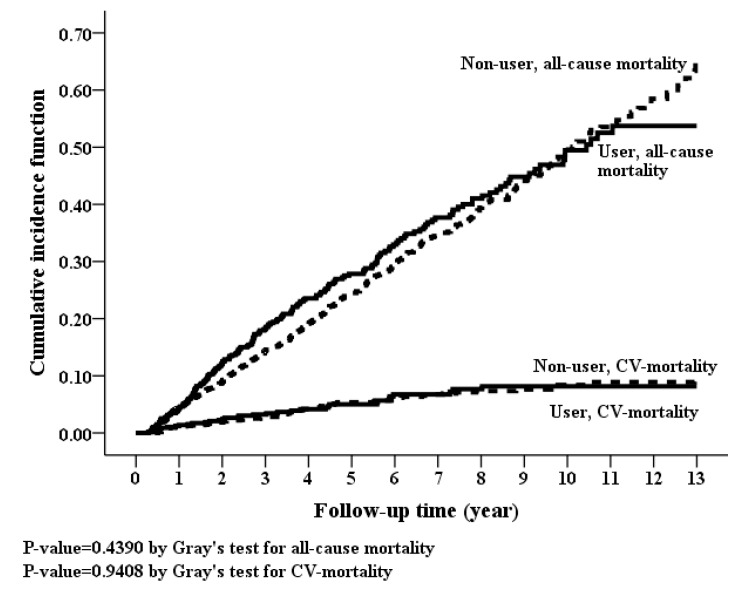
Cumulative incidence rate of all-cause mortality (*p*-value = 0.439, Grey’s test) and cardiovascular disease-mortality (*p*-value = 0.9408, Grey’s test) between spironolactone users and non-users.

Kaplan-Meier curves showed non-significant difference in cumulative incidences for MACE, HHF, CVD mortality and all-cause mortality (Figure 4 and Figure 5). Nonetheless, there were no statistically significant differences in the risks of MACE, HHF, CVD mortality, and all-cause mortality between spironolactone users and non-users, both in the crude models and in adjusted models (Table 2). In addition, spironolactone had no dose-response relationship with MACE, HHF, CVD mortality and all-cause mortality (Table 3).

### 3.5. Subgroup Analysis

**Figure 6:**
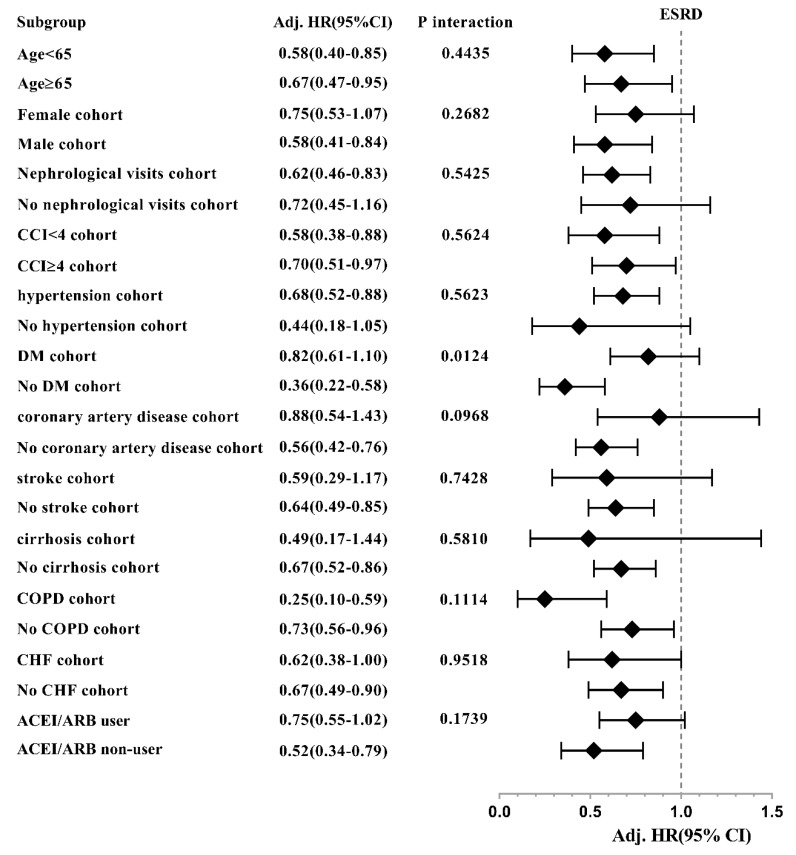
Hazard ratios of end-stage renal disease associated with spironolactone use in subgroup analyses.

**Figure 7:**
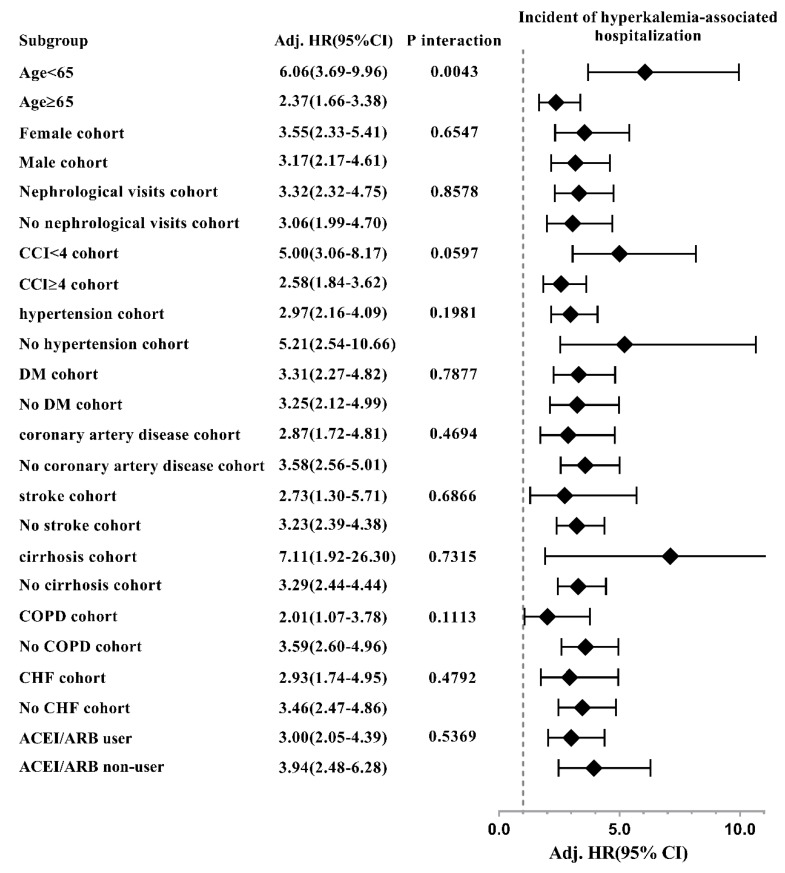
Hazard ratios of progression to hyperkalemia-associated hospitalization associated with spironolactone use in subgroup analyses.

The reduced HRs of ESRD associated with spironolactone use among moderate to severe CKD patients were consistent across most of the patient subgroups, except those with female cohort, no nephrological visit, non-hypertension, DM, stroke, cirrhosis, ACEI/ARB users, while the significant interaction effect was only found between ESRD and DM (Figure 6). Moreover, the increased HRs of HKAH associated with spironolactone use were consistently significant in all the stratified subgroups (Figure 7).

### 3.6. Sensitivity Analyses

The results of a series of sensitivity analyses were shown in Table 4. All the sensitivity tests revealed a lower risk of ESRD and a higher risk of HKAH associated with spironolactone use, indicating the robustness of our findings.

## 4. Discussion

As far as we know, the present study was the first and largest study of nationwide cohort population to assess the effects of spironolactone on patient-centered hard endpoints in patients with moderate to severe (stage 3–4) CKD. The most striking finding was, for the first time, that treatment with spironolactone was associated with a lower risk of ESRD but complicated with a higher risk of HKAH. The dose-dependent effect of spironolactone suggested that a lower risk of ESRD was confounded by a higher risk of HKAH while prescribing higher doses of spironolactone. Insignificant associations were found between spironolactone users and non-users in terms of MACE, HHF, all-cause mortality, and cardiovascular mortality. The associations were further consolidated by the consistent results throughout the sensitivity tests and in most subgroups of patients.

A growing body of clinical evidence has been emerging in support of the independent role of aldosterone in the development and progression in cardiovascular and kidney disease [11,12,13]. The majority of the studies on spironolactone among CKD patients focused on the change in urinary protein/albumin secretion, blood pressure, serum potassium levels and creatinine clearance or estimated glomerular filtration rate in the presence of ACEI and/or ARB treatment [14,15,16,17]. The study duration ranged from 8 to 52 weeks. The patient population was small (*n* = 18–208) with variable methodology and most of them had stage 1–3 CKD. Data were not available on long-term patient-focused outcomes, including cardiovascular events, ESRD, and mortality in any of the trials. Currie et al. demonstrated that combined use of MRA with ACEI and/or ARB significantly lowered proteinuria with a higher risk of hyperkalemia, but was associated with a small, non-significant decline in renal function in a recent meta-analysis [18]. An average increase of 0.19 mmol/L in serum potassium level and three-fold higher risk of hyperkalemia were shown when CKD patients received MRA in addition to ACEI and/or ARB. Our findings were partially in line with those previous studies and extended to clinical endpoints. By using a representative nationwide cohort data with an appropriate follow-up time and 2-to-1 propensity-score matching, we are confident with the reduced risk of ESRD and higher risk of HKAH for spironolactone use in moderate to severe CKD patients. Spironolactone was associated with 34% reduced risk of ESRD and a three times greater risk of HKAH. Therefore, the renoprotective benefit of spironolactone however may be offset by the hyperkalemia risk.

The hyperkalemia risk induced by spironolactone may be mitigated by replacement with nonsteroidal MRA which reportedly had a promising reduction in albuminuria with a lower risk of side effects in patients with diabetic nephropathy [19]. Furthermore, the concomitant use of potassium-lowering agent when prescribing spironolactone and/or other RAAS inhibitors for high-risk patients may also decrease hyperkalemia occurrence. The economic impact of more frequent monitoring of serum potassium concentrations should be evaluated against the beneficial reduction of CKD progression.

Mineralocorticoid receptors (MR) are expressed in podocytes, mesangial cells and endothelial cells and smooth muscle cells of murine vasculature [20]. Renal MR were upregulated in animals models of both type 1 and type 2 DM [21]. In kidney biopsies of patients with renal failure, renal MR mRNA expression in those with heavy albuminuria is 4.6 times higher than those with no albuminuria, microalbuminuria and moderate albuminuria [22]. Aldosterone-mediated renal injury is mainly ascribed to inflammation and fibrosis, independent of the systemic effects on blood pressure. The deleterious effects of aldosterone on the kidneys include glomerular hypertrophy, glomerulosclerosis, proteinuria, and reduced renal blood flow through the upregulation of NADPH oxidase activity, reactive oxygen species, nuclear factor -kβ, pro-inflammatory cytokines and pro-fibrotic proteins [23]. The most convincing evidence for the detrimental role of MR may be derived from the demonstration of MRA, spironolactone or eplerenone, to have renoprotective effects in animal models of kidney diseases. MRA was shown to suppress MR expression, and reduce makers of oxidative stress, pro-inflammatory and pro-fibrotic mediators, including TGF-β, connective tissue growth factor, and osteopontin, in murine models [20,24,25,26,27]. MRA can also attenuate apoptosis and endothelial dysfunction with increases in endothelial nitric oxide synthase [28,29,30]. Further benefits of MRA include improvement of podocyte injury, glomerulosclerosis, proteinuria and glomerular hypertrophy [31,32,33]. Taken together, the reduced risk of ESRD by spironolactone may be ascribed to jointly those favorable hemodynamic changes and metabolic effects of spironolactone.

Recently, Tseng et al. reported the use of spironolactone in advanced (stage 5) CKD patients resulted in higher risks of all-cause mortality, HHF, and infection related mortality and non-significant associations with MACE, CVD mortality and HKAH whereas the impact on ESRD was not evaluated [34]. They attributed the higher mortality risk to the exacerbation of the already present metabolic acidosis, hyperuricemia, endocrinopathy (cortisol, testosterone and glucose homeostasis) and impairment of host immunity after the use of spironolactone. The distinct results from ours may be that our cohort comprised stage 3–4 CKD patients who were not so susceptible to the deleterious metabolic effects by spironolactone. Notably, our intriguing finding of HKAH risk was not present in their stage 5 CKD patients. Potassium balance is usually maintained until reaching stage 5 CKD where more than one-half of patients had mild elevation of potassium. In their study, not only the spironolactone use group but also the non-use group had a higher incidence of HKAH than our groups of spironolactone use and non-use, suggesting that basically stage 5 CKD patients without taking spironolactone even had a higher HKAH risk than stage 3–4 CKD patients with spironolactone treatment. The crude HR of HKAH was not significant between the spironolactone users and non-users in stage 5 CKD patients, whereas a significantly higher risk was seen in stage 3–4 CKD patients treated with spironolactone. Therefore, the effect of spironolactone-induced HKAH was pronounced only in stage 3–4 CKD patients whose HKAH was extremely low without spironolactone use, but not in stage 5 CKD patients who had pre-existing relatively right HKAH even in the absence of spironolactone treatment.

The present study is the first large-scale nationwide one to investigate the effects of spironolactone on several clinical endpoints in stage 3–4 CKD patients. The selection bias was minimized due to the use of medical claim data of NHIRD, which covered more than 99% of Taiwanese residents. In addition, a longer follow-up time and more enrolled patients than previously published trials allowed us to assess the risks and benefits associated with spironolactone in the long run. Furthermore, the robustness of our findings was strengthened by performing propensity-score matching process, sensitivity analyses and subgroup tests. However, some limitations should also be addressed. First, some residual confounding factors may affect our outcomes despite we managed to adjust for clinically important patient characteristics, including socio-economic status, relevant comorbidities and medications usage. The NHIRD also did not contain personal information, which is known as key determinants of clinical outcomes, such as medical adherence, over-the-counter medications, Chinese herbal medicine, tobacco use, and laboratory measurements. A propensity-score matching method was applied to control for the residual confounders. The unmeasured confounders were likely to distribute equally in the spironolactone and control groups. Second, causality cannot be approved in our retrospective observational study. Our national cohort study represented a clinical practice in the real world, unlike the randomized controlled trials where women and high-risk and vulnerable populations are usually excluded. Available evidence also indicated that well-designed observational studies do not systemically overestimate the treatment effects and can yield comparable results to randomized controlled trials [35].

Third, the use status of spironolactone may change over time, biasing the statistical analyses. We defined the spironolactone users by several time intervals and ran the analyses by treating spironolactone use in as-treated model and excluding the control cohort receiving spironolactone during the follow-up period, and checked the cohort effects. All of these tests produced similar results as the primary analyses. Fourthly, there is indication bias inherent to this study because spironolactone was not prescribed with the intention to prevent the progression of CKD. Instead, spironolactone is mainly used as treatment for cirrhosis, resistant hypertension or heart failure and these comorbidities increase the progression of CKD to ESRD. Therefore, the spironolactone users were more frequently co-morbid, especially by cirrhosis, resistant hypertension and heart failure, which was evident from our unmatched data for baseline characteristics. Because the spironolactone users were more comorbid and these comorbidities worsen renal function, the renal protective effect of spironolactone was supposed to augment in the absence of these comorbidities. We also re-ran the multivariate regression analysis using the raw data before propensity score matching and the results were consistent with the primary analyses. Finally, the results of the present study may not be applicable to other population outside Taiwan due to different ethnicity, cultural background and health service systems. It may only apply to stage 3–4 CKD patients, not to advanced (stage 5) CKD.

In conclusion, spironolactone represented a promising treatment option to retard CKD progression to ESRD in patients with moderate to severe (stage 3–4) CKD. The HR of ESRD for spironolactone users versus non-users was 0.66 (0.51–0.84) but it also carried a significantly quantifiable risk of HKAH. There was no significant difference in the risks of MACE, HHF, all-cause mortality and CVD mortality between spironolactone users and non-users. Based on the results of our nationwide population-based cohort study, spironolactone can be prescribed for its renoprotective effect in stage 3–4 CKD but strategic treatments to prevent hyperkalemia should be enforced.

## Figures and Tables

**Table 1:** Baseline characteristics and clinical outcomes of study patients by spironolactone use before and after propensity score matching.

	Before Propensity-Score Matching			After Propensity-Score Matching		
	Non-User	User	*p*-Value	Non-User	User	*p*-Value
Patient number	13,884	785		1386	693	
Age, years	63 ± 16	65 ± 15	<0.001	65 ± 15	65 ± 16	0.814
Gender, Male	7738 (55.73%)	440 (56.05%)	0.862	767 (55.34%)	380 (54.83%)	0.827
Monthly income, New Taiwan Dollars	14,195 ± 14,667	12,283 ± 12,489	<0.001	12,419 ± 12,580	12,397 ± 12,662	0.971
Geographic location						
Northern	6661 (47.98%)	317 (40.38%)	<0.001	532 (38.38%)	279 (40.26%)	0.436
Middle	2396 (17.26%)	207 (26.37%)	<0.001	393 (28.35%)	180 (25.97%)	0.274
Southern	4452 (32.07%)	248 (31.59%)	0.813	439 (31.67%)	223 (32.18%)	0.855
Eastern	375 (2.7%)	13 (1.66%)	0.0968	22 (1.59%)	11 (1.59%)	0.852
Comorbidities within 1 year before the index date						
Hypertension	9192 (66.21%)	584 (74.39%)	<0.001	1033 (74.53%)	514 (74.17%)	0.859
Diabetes	5533 (39.85%)	371 (47.26%)	<0.001	666 (48.05%)	326 (47.04%)	0.664
Coronary artery disease	2665 (19.19%)	231 (29.43%)	<0.001	398 (28.72%)	195 (28.14%)	0.783
Stroke	1924 (13.86%)	135 (17.2%)	0.009	258 (18.61%)	114 (16.45%)	0.225
Atrial fibrillation	300 (2.16%)	44 (5.61%)	<0.001	64 (4.62%)	34 (4.91%)	0.770
Cirrhosis	202 (1.45%)	72 (9.17%)	<0.001	74 (5.34%)	36 (5.19%)	0.890
PAOD	267 (1.92%)	23 (2.93%)	0.049	38 (2.74%)	21 (3.03%)	0.709
Cancer	801 (5.77%)	57 (7.26%)	0.083	82 (5.92%)	46 (6.64%)	0.519
COPD	2015 (14.51%)	166 (21.15%)	<0.001	298 (21.5%)	144 (20.78%)	0.705
CHF	1359 (9.79%)	209 (26.62%)	<0.001	359 (25.9%)	172 (24.82%)	0.594
Charlson comorbidity index	3.1 ± 2.4	4 ± 2.6	<0.001	3.8 ± 2.5	3.8 ± 2.5	0.826
Anti-hypertensive drugs						
ACEI/ARB	6030 (43.43%)	417 (53.12%)	<0.001	727 (52.45%)	358 (51.66%)	0.733
α-blocker	1393 (10.03%)	90 (11.46%)	0.195	158 (11.4%)	79 (11.4%)	1.000
β--blocker	4828 (34.77%)	325 (41.4%)	<0.001	546 (39.39%)	278 (40.12%)	0.751
Calcium channel blocker						
Non-DHP	1439 (10.36%)	118 (15.03%)	<0.001	213 (15.37%)	105 (15.15%)	0.897
DHP	5544 (39.93%)	349 (44.46%)	0.012	628 (45.31%)	311 (44.88%)	0.852
Other Diuretics						
Thiazide	2298 (16.55%)	181 (23.06%)	<0.001	313 (22.58%)	147 (21.21%)	0.478
Loop diuretics	1623 (11.69%)	246 (31.34%)	<0.001	343 (24.75%)	179 (25.83%)	0.592
Miscellaneous	670 (4.83%)	62 (7.9%)	<0.001	106 (7.65%)	50 (7.22%)	0.724
Antidiabetic medication						
Sulfonylurea	3943 (28.4%)	277 (35.29%)	<0.001	479 (34.56%)	241 (34.78%)	0.922
Meglitinide	685 (4.93%)	51 (6.5%)	0.051	88 (6.35%)	41 (5.92%)	0.700
α-glucosidase inhibitor	998 (7.19%)	79 (10.06%)	0.003	147 (10.61%)	70 (10.1%)	0.723
Biguanide	3611 (26.01%)	249 (31.72%)	<0.001	437 (31.53%)	221 (31.89%)	0.868
Thiazolidinedione	1013 (7.3%)	70 (8.92%)	0.091	111 (8.01%)	63 (9.09%)	0.401
Insulin	937 (6.75%)	87 (11.08%)	<0.001	146 (10.53%)	69 (9.96%)	0.684
Statins	3724 (26.82%)	245 (31.21%)	0.007	430 (31.02%)	216 (31.17%)	0.947
Aspirin	3753 (27.03%)	274 (34.9%)	<0.001	492 (35.5%)	236 (34.05%)	0.516
NSAIDs	2095 (15.09%)	149 (18.98%)	0.003	257 (18.54%)	126 (18.18%)	0.841
NaHCO3	147 (1.06%)	10 (1.27%)	0.569	13 (0.94%)	8 (1.15%)	0.642
Nephrology visit within 1 year before the index date	1.4 ± 2.1	1.3 ± 2.1	0.243	1.3 ± 2	1.3 ± 2.1	0.994
Propensity score	0.05 ± 0.06	0.14 ± 0.17	<0.001	0.1 ± 0.11	0.1 ± 0.11	0.999
Outcome						
ESRD	2399 (17.28%)	102 (12.99%)	0.002	266 (19.19%)	88 (12.7%)	<0.001
MACEs	1084 (7.81%)	63 (8.03%)	0.878	123 (8.87%)	56 (8.08%)	0.599
Hospitalization for heart-failure	423 (3.05%)	36 (4.59%)	0.021	76 (5.48%)	29 (4.18%)	0.243
Hyperkalemia-associated hospitalization	724 (5.21%)	151 (19.24%)	<0.001	92 (6.64%)	123 (17.75%)	<0.001
Mortality	2857 (20.58%)	226 (28.79%)	<0.001	386 (27.85%)	192 (27.71%)	0.9448
CVD death	413 (3.0%)	39 (5.0%)	0.002	70 (5.1%)	34 (4.9%)	0.972

Abbreviations: PAOD, peripheral arterial occlusion disease; COPD, chronic obstructive pulmonary disease; CHF, congestive heart failure; ACEI, angiotensin-converting enzyme inhibitor; ARB, angiotensin II receptor blocker; DHP, dihydropyridine; NSAID, Non-Steroidal Anti-Inflammatory Drug; ESRD, end-stage renal disease; MACE, major adverse cardiovascular events; CVD, cardiovascular disease.

**Table 2:** Risks for ESRD, MACEs, hospitalization for heart-failure, hyperkalemia-associated hospitalization and mortality among patients with stage 3–4 CKD by spironolactone use.

	Users		Non-Users		Users Compared to Non-Users			
	Event	IR (95% CI)	Event	IR (95% CI)	Crude HR (95% CI)	*p*-Value	Adjusted HR ^†^ (95% CI)	*p*-Value
ESRD	88	39.2 (31.01–47.39)	266	53.69 (47.24–60.14)	0.65 (0.51–0.83)	<0.001	0.66 (0.51–0.84)	<0.001
MACE ^§^	56	24.94 (18.41–31.48)	123	24.83 (20.44–29.21)	0.93 (0.68–1.27)	0.647	0.93 (0.67–1.28)	0.647
Hospitalization for heart-failure	29	12.92 (8.22–17.62)	76	15.34 (11.89–18.79)	0.77 (0.50–1.19)	0.238	0.77 (0.50–1.18)	0.225
Hyperkalemia-associated hospitalization	123	54.79 (45.1–64.47)	92	18.57 (14.77–22.36)	2.98 (2.28–3.90)	<0.001	3.17 (2.41–4.17)	<0.001
All-cause mortality	192	64.42 (55.31–73.53)	386	60.47 (54.44–66.5)	1.07 (0.90–1.27)	0.432	1.10 (0.92–1.30)	0.294
Cardiovascular death	34	11.41 (7.57–15.24)	70	10.97 (8.4–13.53)	1.02 (0.67–1.53)	0.941	1.14 (0.75–1.74)	0.533

Abbreviation: CI, confidence interval; HR, hazard ratio; IR, incidence rate (per 1000 person-years);.ESRD, end-stage renal disease; MACE, major adverse cardiovascular events. ^†^ Adjusted for all covariates in Table 1 after propensity-score matching. ^§^ MACE, the composite of acute myocardial infarction and ischemic stroke.

**Table 3:** Risks for ESRD, MACEs, HHF, HKAH and mortality among patients with stage 3–4 CKD by prescribed daily dose and cumulative defined daily dose of spironolactone within 90 days.

	Outcomes											
	ESRD		MACE ^§^		HHF		HKAH		All-Cause Mortality		CVD Mortality	
	Adj. HR ^†^ (95% CI)	*p*-Value	Adj. HR ^†^ (95% CI)	*p*-Value	Adj. HR ^†^ (95% CI)	*p*-Value	Adj. HR ^†^ (95% CI)	*p*-Value	Adj. HR ^†^ (95% CI)	*p*-Value	Adj. HR ^†^ (95% CI)	*p*-Value
Prescribed daily dose (mg)												
Spironolactone (vs. non-use)												
<12.5 mg	0.73 (0.52–1.00)	0.0523	0.97 (0.64–1.45)	0.8688	0.78 (0.43–1.41)	0.4102	2.81 (1.96–4.03)	<0.0001	1.15 (0.91–1.45)	0.2304	0.97 (0.54–1.71)	0.904
12.5–25 mg	0.59 (0.39–0.91)	0.0175	0.57 (0.29–1.11)	0.0965	0.90 (0.45–1.81)	0.7732	2.74 (1.8–4.17)	<0.0001	1.15 (0.87–1.53)	0.3302	1.34 (0.73–2.46)	0.3437
≥25 mg	0.57 (0.35–0.91)	0.0193	1.34 (0.79–2.25)	0.2803	0.61 (0.26–1.44)	0.2572	4.80 (3.3–6.97)	<0.0001	0.95 (0.70–1.29)	0.744	0.87 (0.41–1.84)	0.7107
*p*-trend		0.0057		0.2428		0.5971		<0.0001		0.4920		0.7568
Cumulative defined daily dose (cDDD) (vs. non-use)												
Spironolactone												
≤30 cDDD	0.68 (0.52–0.89)	0.005	0.82 (0.57–1.19)	0.2951	0.84 (0.53–1.34)	0.4749	2.77 (2.04–3.75)	<0.0001	1.16 (0.96–1.4)	0.1312	1.09 (0.69–1.7)	0.7206
>30 cDDD	0.60 (0.37–0.97)	0.0353	1.36 (0.80–2.33)	0.2600	0.53 (0.20–1.37)	0.1903	4.70 (3.21–6.87)	<0.0001	0.94 (0.68–1.28)	0.6708	0.89 (0.42–1.9)	0.7695
*p*-trend		0.0038		0.2588		0.3522		<0.0001		0.2535		0.9644

Abbreviation: Adj. HR = Adjusted Hazard Ratio; CI, confidence interval; ESRD, end-stage renal disease; MACE, major adverse cardiovascular events; HHF, hospitalization for heart-failure, HKAH, hyperkalemia-associated hospitalization. ^†^ Adjusted for all covariates in Table 1 after propensity-score matching. ^§^ MACE, the composite of acute myocardial infarction and ischemic stroke.

**Table 4:** Sensitivity Analyses.

	Outcomes											
	ESRD		MACE ^§^		HHF		HKAH		All-Cause Mortality		CVD-Mortality	
	Adj. HR ^†^ (95% CI)	*p*-Value	Adj. HR ^†^ (95% CI)	*p*-Value	Adj. HR ^†^ (95% CI)	*p*-Value	Adj. HR ^†^ (95% CI)	*p*-Value	Adj. HR ^†^ (95% CI)	*p*-Value	Adj. HR ^†^ (95% CI)	*p*-Value
**Time intervals for defining Spironolactone users/non-users**												
Within 60 days												
Spironolactone use (vs. non-use)	0.59 (0.46–0.77)	<0.0001	1.08 (0.77–1.50)	0.6618	0.82 (0.53–1.28)	0.3861	2.40 (1.82–3.16)	<0.0001	1.09 (0.91–1.30)	0.3495	1.20 (0.80–1.82)	0.3785
Within 120 days												
Spironolactone use (vs. non-use)	0.73 (0.58–0.92)	0.0088	0.96 (0.70–1.32)	0.8003	1.12 (0.74–1.71)	0.5816	2.52 (1.94–3.26)	<0.0001	1.01 (0.85–1.19)	0.9148	1.12 (0.18–7.09)	0.904
Within 180 days												
Spironolactone use (vs. non-use)	0.69 (0.55–0.87)	0.0019	0.95 (0.68–1.32)	0.7508	0.84 (0.54–1.3)	0.4367	3.08 (2.28–4.16)	<0.0001	1.01 (0.85–1.21)	0.8976	1.00 (0.62–1.61)	0.9847
**Exclude control cohort receiving spironolactone during the follow-up period**												
Spironolactone use (vs. non-use)	0.63 (0.49–0.81)	0.0002	0.91 (0.66–1.26)	0.5724	0.76 (0.49–1.19)	0.2263	3.65 (2.71–4.91)	<0.0001	1.07 (0.89–1.27)	0.4773	1.11 (0.70–1.78)	0.6565
**Consider spironolactone status change as censored (As-treat model)**												
Spironolactone use (vs. non-use)	0.37 (0.21–0.64)	0.0004	0.71 (0.44–1.14)	0.1553	1.11 (0.65–1.92)	0.6967	9.18 (6.69–12.6)	<0.0001	1.17 (0.83–1.66)	0.3642	1.42 (0.68–2.97)	0.3571
**Cohort in 2000–2006**												
Spironolactone use (vs. non-use)	0.48 (0.28–0.81)	0.0064	1.17 (0.60–2.28)	0.6515	1.02 (0.24–4.27)	0.9828	2.38 (1.3–4.35)	0.0048	1.34 (0.76–2.37)	0.3125	1.34 (0.35–5.07)	0.6672
Cohort in 2007–2013												
Spironolactone use (vs. non-use)	0.71 (0.53–0.94)	0.0184	0.88 (0.61–1.27)	0.5035	0.73 (0.43–1.21)	0.2167	3.33 (2.45–4.52)	<0.0001	1.18 (0.98–1.43)	0.0818	1.16 (0.74–1.82)	0.5137
**Using raw data (before propensity-score matching) as analyzed data**												
Spironolactone use (vs. non-use)	0.65 (0.53–0.80)	<0.0001	1.02 (0.78–1.33)	0.9091	0.85 (0.59–1.21)	0.3649	3.00 (2.46–3.67)	<0.0001	1.21 (1.04–1.40)	0.0141	1.23 (0.27–5.62)	0.7899

Abbreviation: Adj. HR = Adjusted Hazard Ratio; CI, confidence interval; ESRD, end-stage renal disease; MACE, major adverse cardiovascular events; HHF, hospitalization for heart-failure, HKAH, hyperkalemia-associated hospitalization. **^†^** Adjusted for all covariates in Table 1 after propensity-score matching. ^§^ MACE, the composite of acute myocardial infarction and ischemic stroke.
